# Ecological Importance of Large-Diameter Trees in a Temperate Mixed-Conifer Forest

**DOI:** 10.1371/journal.pone.0036131

**Published:** 2012-05-02

**Authors:** James A. Lutz, Andrew J. Larson, Mark E. Swanson, James A. Freund

**Affiliations:** 1 College of the Environment, University of Washington, Seattle, Washington, United States of America; 2 Department of Forest Management, University of Montana, Missoula, Montana, United States of America; 3 School of the Environment, Washington State University, Pullman, Washington, United States of America; 4 School of Environmental and Forest Sciences, University of Washington, Seattle, Washington, United States of America; DOE Pacific Northwest National Laboratory, United States of America

## Abstract

Large-diameter trees dominate the structure, dynamics and function of many temperate and tropical forests. Although both scaling theory and competition theory make predictions about the relative composition and spatial patterns of large-diameter trees compared to smaller diameter trees, these predictions are rarely tested. We established a 25.6 ha permanent plot within which we tagged and mapped all trees ≥1 cm dbh, all snags ≥10 cm dbh, and all shrub patches ≥2 m^2^. We sampled downed woody debris, litter, and duff with line intercept transects. Aboveground live biomass of the 23 woody species was 507.9 Mg/ha, of which 503.8 Mg/ha was trees (SD = 114.3 Mg/ha) and 4.1 Mg/ha was shrubs. Aboveground live and dead biomass was 652.0 Mg/ha. Large-diameter trees comprised 1.4% of individuals but 49.4% of biomass, with biomass dominated by *Abies concolor* and *Pinus lambertiana* (93.0% of tree biomass). The large-diameter component dominated the biomass of snags (59.5%) and contributed significantly to that of woody debris (36.6%). Traditional scaling theory was not a good model for either the relationship between tree radii and tree abundance or tree biomass. Spatial patterning of large-diameter trees of the three most abundant species differed from that of small-diameter conspecifics. For *A. concolor* and *P. lambertiana*, as well as all trees pooled, large-diameter and small-diameter trees were spatially segregated through inter-tree distances <10 m. Competition alone was insufficient to explain the spatial patterns of large-diameter trees and spatial relationships between large-diameter and small-diameter trees. Long-term observations may reveal regulation of forest biomass and spatial structure by fire, wind, pathogens, and insects in Sierra Nevada mixed-conifer forests. Sustaining ecosystem functions such as carbon storage or provision of specialist species habitat will likely require different management strategies when the functions are performed primarily by a few large trees as opposed to many smaller trees.

## Introduction

Large-diameter trees dominate the structure, dynamics, and function of many temperate and tropical forest ecosystems and are of considerable scientific and social interest. They comprise a large fraction of forest wood volume, biomass and carbon stocks [1], [2], and modulate stand-level leaf area, transpiration, and microclimates [3], [4]. Large-diameter trees contribute disproportionately to reproduction [5], influence the rate and pattern of tree regeneration and forest succession [6], and originate further disturbance by crushing or injuring neighboring trees when they fall to the ground [7], [8]. Arboreal wildlife species preferentially occupy large trees as habitat (e.g. [9]), and the greater structural complexity of large tree crowns [10] supports habitat for obligate wildlife species (e.g. [11]), unique epiphyte communities [12], and soil development and water storage within the forest canopy [13].

Large-diameter trees continue to contribute disproportionately to forest ecosystem structure and function after they die. Dead large-diameter trees persist as standing snags for many years, providing additional wildlife habitat. In temperate forests large-diameter logs may persist on the forest floor for centuries, where they continue to provide habitat for diverse assemblages of vertebrates and invertebrates and microorganisms, store carbon and other nutrients, serve as substrates for tree regeneration, and play numerous other functional roles [14], [15].

Human societies derive many non-timber values from large-diameter trees. Tree ring chronologies from large trees provide long records of past forest development and disturbance [16], as well as proxy records of annual climatic variation [17]: they are an important source of the data required to test and refine ecological theories and models. Large trees are culturally [18] and spiritually important [19] in many societies; individuals and organizations maintain large tree registries (e.g., [20]), and government agencies manage parks and preserves dedicated to the conservation of exceptionally large trees, such as Redwood and Sequoia & Kings Canyon National Parks in California, USA.

Populations of large-diameter trees can be intractable study subjects. Large-diameter trees occur at low densities and estimates of their abundance, spatial patterns, and contributions to ecosystem function (e.g. biomass) are subject to high rates of sampling error [21], [22]. Consequently, descriptive statistics and hypothesis tests for large-diameter trees require very large sample plots [22]. The combination of low abundance and low mortality rates [8], [23], [24] make detecting changes in demographic rates or spatial patterns of large-diameter trees even more difficult, further underscoring the requirement for large sample plots. Conventional studies based on small (1 ha to 4 ha) plots often do not contain enough large-diameter trees to conduct even community-level (i.e., pooled across species) analyses (e.g., [24]). Consequently, despite their ecological and cultural significance, relatively less is known – and with greater uncertainty – about the abundance, distribution, and dynamics of large-diameter trees.

### Predictions for large-diameter trees

Scaling theory and competition theory both provide frameworks for predictions about the relative contributions of large-diameter structures to aboveground biomass, the spatial distribution of large-diameter trees, and the spatial relationships between large-diameter and small-diameter trees. Scaling theory predicts that a relatively few trees in the largest diameter classes will dominate stand-level aboveground biomass [25], [26], and that there are continuous relationships between tree diameter and density, and total forest biomass. However, scaling theory has been repeatedly shown to underpredict large tree densities and mortality rates [27], [28]. This discrepancy likely arises because trees rarely die from competition once they reach large sizes but rather succumb to biological agents, physical disturbances, and combinations thereof [8], [29]. Although scaling theory predicts dominance of biomass pools by a few large individuals, the simplifying assumptions about tree mortality embedded in the theory may render it inadequate to predict accurately either the aggregate large tree contributions to stand biomass or the local-scale variation. We were interested in quantifying the actual contribution of large-diameter pieces to aboveground biomass pools—which should be substantial [14]—because predictions from scaling theory alone may not be accurate enough to serve as inputs into ecosystem models or to support sound natural resource policies and management.

Tree spatial patterns integrate past tree-tree interactions. Competition theory predicts that distance and density-dependent growth and mortality during forest development will lead to increasingly uniform spatial patterns in larger diameter classes [30], [31]. Therefore, the arrangement of large-diameter trees should be more uniform than small-diameter trees, and the largest trees should exhibit spatial regularity at the tree neighborhood scale. Competition theory also predicts spatial relationships between large and small-diameter trees. When large trees compete asymmetrically with small trees their respective spatial locations become segregated because seedlings preferentially survive and grow into understory trees where they are not suppressed by larger competitors [30], [32], [33].

Our study was motivated by three purposes: (1) determine the degree to which predictions from ecological theory hold for contemporary populations of large-diameter trees; (2) establish a permanent forest research plot of sufficient size to detect and attribute forest ecosystem change, including for the large-diameter component, in order to test future predictions against longitudinal data; and (3) support current management efforts to restore large-diameter tree populations in Sierra Nevada mixed-conifer forest, which were dramatically reduced by widespread logging throughout the range of this important forest type during the 19^th^ and 20^th^ centuries [34], [35]. We established the Yosemite Forest Dynamics Plot (YFDP) in an old-growth Sierra Nevada mixed-conifer forest and within the plot quantified the relative contribution of large-diameter trees, snags, and down woody debris to the aboveground biomass pools, the comparative spatial patterns of large-diameter and small-diameter trees, and spatial relationships between them.

## Results

### Species composition

**Figure 1:**
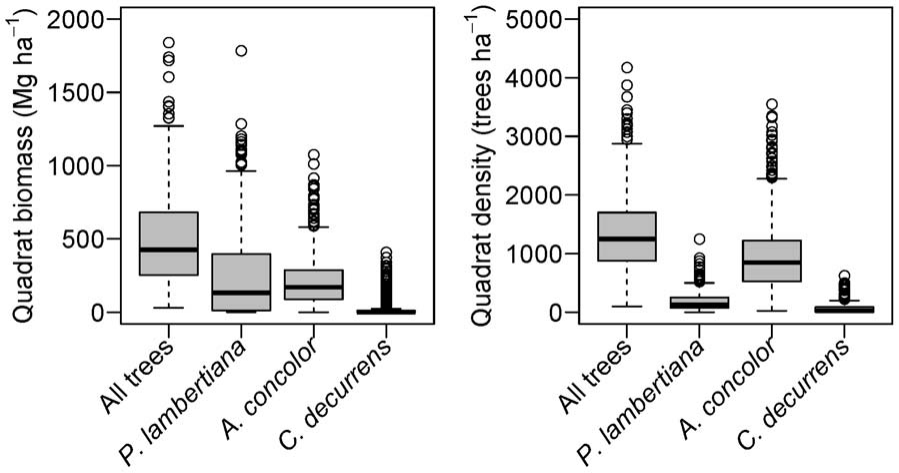
Heterogeneity in biomass and density of the principal tree species of the Yosemite Forest Dynamics Plot. Each boxplot represents values from the 640, 20 m×20 m quadrats of the plot.

**Figure 2:**
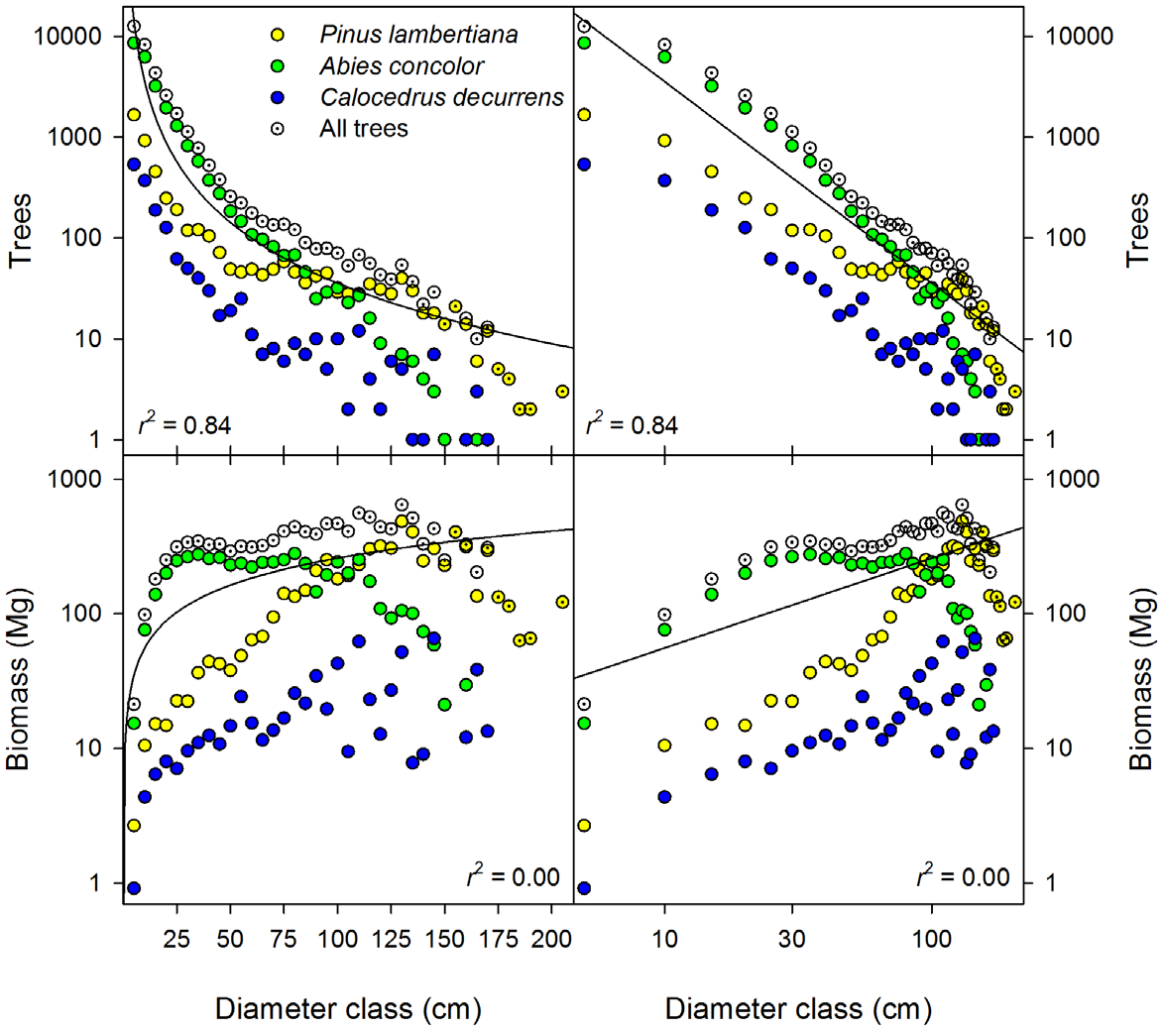
Diameter distribution of the number of trees and the biomass of trees in the Yosemite Forest Dynamics Plot. Each point represents a 5 cm diameter class (first bin; 1 cm≤dbh<5 cm) of the trees from the entire 25.6 ha plot (34,458 live stems ≥1 cm dbh totaling 12,897 Mg); identical data are shown with linear diameter bins (left) and log diameter (right). Solid lines represent the best fitting equation of the form specified by scaling theory, 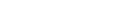 (r^2^ = 0.84) and 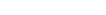 (r^2^ = 0.00), where *r* is tree radius.

**Figure 3:**
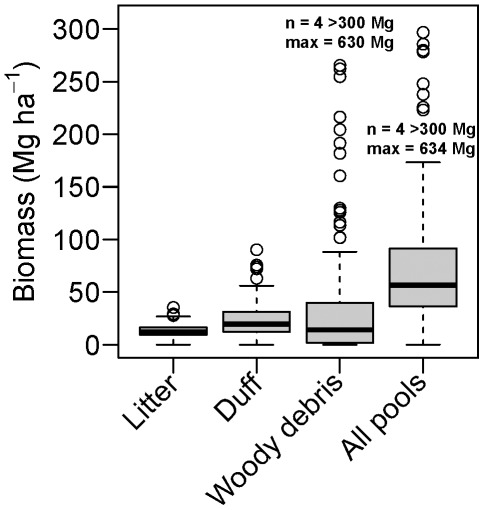
Biomass of forest floor components of the Yosemite Forest Dynamics Plot. Each boxplot represents values from 112 transects of 20 m (2.24 km of line transects). Outliers represent intercepted pieces of large-diameter debris.

In the 25.6 ha of the Yosemite Forest Dynamics Plot (YFDP), there were 34,458 live stems ≥1 cm dbh of 11 tree species (Table 1) and 3.87 ha (15.1%) of continuous shrub cover comprising 12 shrub species that reach 1 cm in diameter at 1.37 m height (Table 2). Eleven plant families were represented. All woody stems were native plants. Live tree basal area was 64.3 m^2^/ha and biomass was 503.8 Mg/ha (SD = 114.3 Mg/ha) (Table 3). Of the three principal species by biomass (*Pinus lambertiana, Abies concolor*, and *Calocedrus decurrens*), *P. lambertiana* had a much higher average biomass (Fig. 1) and exhibited a rotated-sigmoid diameter distribution, possibly reflecting lower mortality of middle-aged individuals (Fig. 2). Diameter distributions of *A. concolor* and *C. decurrens* followed negative exponential distributions (Fig. 2). Relative dominance of *Abies concolor* declines at diameters above ∼90 cm (Fig. 2). *Calocedrus decurrens* exhibits almost an order of magnitude less biomass than either *P. lambertiana* or *A. concolor* (Fig. 2). However, some individuals do persist into large diameter classes (Fig. 2). Live shrub biomass was 4.1 Mg/ha (Table 2). There were 2,697 snags (19.9% of living trees of this diameter). Biomass of snags ≥10 cm dbh was 43.0 Mg/ha (Table 3). Biomass of the forest floor components (Table 3, Fig. 3) was 53.1 Mg/ha for down woody debris (SD = 102.9 Mg/ha) and 48.0 Mg/ha (SD = 22.5) for fine fuels (Table 3). Litter and duff averaged 1.05 cm (SD = 0.38) and 1.20 cm (SD = 0.68) in depth, respectively. A correlogram analysis of woody debris volumes as estimated by the 20 m line intercept segments showed no spatial correlation in fuel loads at any distance. Total above-ground biomass of living and dead components was 652.0 Mg/ha.

**Table 1:** Tree species within the Yosemite Forest Dynamics Plot in 2010.

Tree species	Family	Density (stems/ha)	Basal area (m^2^/ha)	Stems ≥1 cm dbh	Stems ≥10 cm dbh	Stems ≥100 cm dbh	Large-diameter prop. (%)
Trees ≥1 cm dbh							
*Abies concolor*	Pinaceae	956.3	29.28	24,481	9,634	103	0.4
*Pinus lambertiana*	Pinaceae	185.5	28.75	4,748	2,166	339	7.1
*Cornus nuttallii*	Cornaceae	92.5	0.26	2,368	287	-	-
*Calocedrus decurrens*	Cupressaceae	62.2	4.78	1,592	685	45	2.8
*Quercus kelloggii*	Fagaceae	43.3	1.12	1,109	735	-	-
*Prunus* spp.	Rosaceae	5.0	t	128	-	-	-
*Abies magnifica*	Pinaceae	0.4	0.06	11	5	1	9.1
*Salix scouleriana*	Salicaceae	0.4	t	11	-	-	-
*Pseudotsuga menziesii*	Pinaceae	0.2	0.03	6	3	1	16.7
*Pinus ponderosa*	Pinaceae	t	0.01	2	1	-	-
*Rhamnus californica*	Rhamnaceae	t	t	1	-	-	-
Live tree total		1,346.0	64.32	34,458	13,516	489	1.4
Snags ≥10 cm dbh							
*Abies concolor*					1,971	64	3.2
*Pinus lambertiana*					530	133	25.1
*Quercus kelloggii*					127	-	-
*Calocedrus decurrens*					46	5	10.9
*Pseudotsuga menziesii*					1	1	100.0
*Cornus nuttallii*					1	-	-
Unknown					21	7	33.3
Dead tree total					2,697	210	7.8

t – trace; less than one tree per 10 ha; less than 0.01 m^2^/ha.

**Table 2:** Shrub species occurring in patches of continuous cover ≥2 m^2^ within the Yosemite Forest Dynamics Plot in 2010.

Species	Family	Cover (m^2^)	Demography plot data		YFDP extrapolation	
			Density^a^ (stems/m^2^)	Biomass (kg/m^2^)	Density (stems/ha)	Biomass (Mg/ha)
*Arctostaphylos patula*	Ericaceae	2,524	5.333	14.747	526	1.454
*Ceanothus cordulatus*	Rhamnaceae	1,220	1.667	1.189	79	0.057
*Ceanothus integerrimus*	Rhamnaceae	194	7.875	10.427	60	0.079
*Ceanothus parvifolius*	Rhamnaceae	187	3.250	1.527	24	0.011
*Chrysolepis sempervirens*	Fagaceae	13,082	3.167	1.464	1,618	0.748
*Corylus cornuta* var. *californica*	Betulaceae	13,310	1.000	1.565	520	0.814
*Cornus serecia*	Cornaceae	2,320	8.667	6.087	785	0.552
*Leucothoe davisiae*	Ericaceae	2,151	0.250	2.430	21	0.204
*Vaccinium uliginosum*	Ericaceae	2,937	0.083	1.069	10	0.123
*Sambucus racemosa* b	Adoxaceae	13	1.000	1.565	t	0.001
*Rhododendron occidentale* c	Ericaceae	687	0.083	1.069	2	0.029
*Ribes nevadense* c	Grossulariaceae	7	0.083	1.069	t	t
*Ribes roezlii* d	Grossulariaceae	66	0	0.534	0	0.001
Total		38,698			3,645	4.103

Baseline density and biomass equations were generated from 25, 2 m×2 m shrub demography plots, and allometric equations from [90].

a Stems ≥1 cm dbh.

b Substituted biomass and density for *Corylus cornuta* var. *californica*.

c Substituted biomass and density for *Vaccinium uliginosum*.

d Substituted one half the biomass of *Vaccinium uliginosum*. No stems reach 1 cm dbh.

t – trace; <1 stem/ha; <1 kg/ha.

**Table 3:** Biomass within the Yosemite Forest Dynamics Plot in 2010.

Tree species	Biomass ≥1 cm (Mg/ha)		Biomass ≥10 cm (Mg/ha)		Biomass ≥100 cm (Mg/ha)		Large-diameter prop. (%)
Trees ≥1 cm							
*Abies concolor*	214.703	(37.505)	210.533	(36.916)	47.983	(8.950)	22.3
*Pinus lambertiana*	254.039	(66.623)	253.380	(66.508)	187.345	(47.594)	73.7
*Cornus nuttallii*	1.411	(0.301)	0.765	(0.199)	-	-	-
*Calocedrus decurrens*	24.978	(7.911)	24.764	(7.845)	12.964	(4.076)	51.9
*Quercus kelloggii*	7.849	(1.935)	7.736	(1.907)	-	-	-
*Prunus* spp.	0.005	(0.002)	-	-	-	-	-
*Abies magnifica*	0.609	(0.110)	0.609	(0.110)	0.469	(0.078)	77.0
*Salix scouleriana*	t	t	-	-	-	-	-
*Pseudotsuga menziesii*	0.146	(0.033)	0.144	(0.032)	0.134	(0.030)	91.8
*Pinus ponderosa*	0.064	(0.003)	0.064	(0.003)	-	-	-
*Rhamnus californica*	t	t	-	-	-	-	-
Live tree total	503.804	(114.346)	497.994	(113.444)	248.896	(60.651)	49.4
Snags ≥10 cm							
*Abies concolor*			20.276		6.708		33.1
*Pinus lambertiana*			21.167		17.959		84.8
*Quercus kelloggii*			0.244		-		-
*Calocedrus decurrens*			0.893		0.551		61.7
*Pseudotsuga menziesii*			0.196		0.196		100.0
*Cornus nuttallii*			t		-		-
Unknown			0.181		0.147		81.2
Dead tree total			42.958		25.562		59.5
Forest floor woody debris ≥10 cm			53.099	(102.897)	19.444	(78.977)	36.6
Shrubs total	4.103		-		-		-
Forest floor fine fuels†							
100-hour fuels	4.562	(4.820)	-		-		-
10-hour fuels	5.176	(3.487)	-		-		-
1-hour fuels	1.129	(0.834)	-		-		-
Litter	13.150	(6.244)	-		-		-
Duff	24.017	(16.517)	-		-		-
Total fine fuels	48.034	(22.495)	-		-		-

Biomass is shown to three significant figures (corresponding to 1 kg/ha) to facilitate comparison between less abundant, small-diameter species and more abundant species (standard deviation shown in parentheses). Standard deviation of tree biomass was based on the root mean squared error of the underlying allometric equations, and standard deviation of down woody debris biomass was based on Brown's method [89]. Biomass of shrubs and snags are derived from cover (m^2^) or measured dimensions and fixed wood density values [see Methods]. Total of living and dead biomass pools was 652.0 Mg/ha.

t – trace; less than 1 kg/ha.

† Fine litter measured by fuel classifications [88]. 100-hour fuels are defined as twigs and fragments with diameter 1″ to 3″ (2.5 cm to 7.6 cm); 10-hour fuels have diameter 0.25″ to 1″ (0.6 cm to 2.5 cm); 1-hour fuels have diameter 0″ to 0.25″ (0 cm to 0.6 cm). Litter and duff are measured by depth.

### Large-diameter composition

The large diameter component dominated most biomass pools (Table 3). For living trees, 1.4% of individuals had dbh ≥100 cm dbh (19.1 large-diameter trees ha^−1^), but these individuals comprised 49.4% of tree biomass. For snags, 12.4% were large-diameter, comprising 59.5% of snag biomass. Snags ≥100 cm dbh were about half as numerous (42.9%) as live trees ≥100 cm dbh. There were 10 pieces of woody debris ≥100 cm (3.8%) measured on the line intercepts, and the large debris component comprised 36.6% of down woody debris biomass. There is, by definition, no large-diameter component to shrubs, fine fuels, litter or duff. Overall, large-diameter structures constituted 44.9% of above-ground live and dead biomass.

Scaling theory was informative for the relationship between tree density and diameter class (r^2^ = 0.84); the theoretical relationship under-predicted the density of medium and large trees, but over-predicted the density of trees ≥170 cm dbh (Fig. 2). Although informative, the relationship between tree density and diameter class was better explained by a negative exponential distribution (r^2^ = 0.99). The theoretical relationship between tree radii and biomass was not informative (r^2^ = 0.00) (Fig. 2).

### Spatial patterns

**Figure 4:**
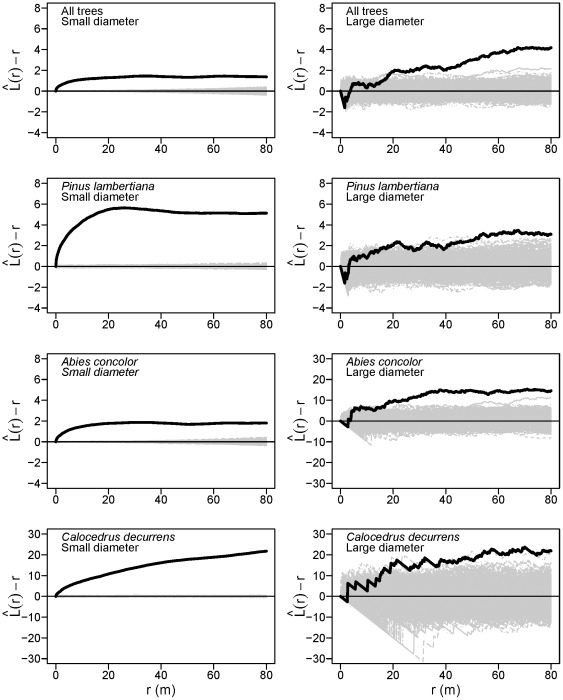
Univariate tree spatial patterns in the Yosemite Forest Dynamics Plot. Solid black lines show the 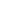 statistic for the actual patterns, where *r* is the intertree distance; thin gray lines show 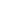 curves for 999 simulations of complete spatial randomness. Positive values indicate spatial clumping and negative values indicate spatial regularity. Large-diameter trees are ≥100 cm dbh; small-diameter trees are <100 cm dbh.

Small-diameter subpopulations of *A. concolor*, *C. decurrens*, *P. lambertiana*, as well as all tree species combined, exhibited significant aggregation relative to the null model of complete spatial randomness (CSR) from 0–9 m (Monte Carlo goodness-of-fit tests; *A. concolor*: *P* = 0.004; *C. decurrens*: *P* = 0.001; *P. lambertiana*: *P* = 0.001, all trees: *P* = 0.004). In other words, when averaged across all points in a given pattern, small-diameter trees of these species have more neighbors of the same type located within a circle with a radius of 9 m than would be expected if tree locations were completely independent of each other. 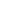 values for small-diameter stems of both *P. lambertiana* and *A. concolor* rose steeply at small scales (Fig. 4), reaching a plateau at about 20 m, indicating that the strong spatial aggregation in these respective subpopulations primarily manifests at scales <20 m. The 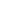 curves for small-diameter *C. decurrens* stems rose steadily from 0–80 m, indicating moderate but consistent clustering across the entire range of scales analyzed (Fig. 4).

The spatial arrangement of large-diameter *A. concolor*, *C. decurrens* and *P. lambertiana* individually, and for all species combined, were not different from complete spatial randomness from 0–9 m (Monte Carlo goodness-of-fit tests; *A. concolor*: *P* = 0.012; *C. decurrens*: *P* = 0.074; *P. lambertiana*: *P* = 0.132; all trees: *P* = 0.057). However, the behavior of the individual 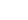 curves from 0–80 m reveals spatial structure within large-diameter *A. concolor* and *P. lambertiana* subpopulations at other interpoint distances (Fig. 4). The empirical 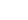 curve (Fig. 4) for large-diameter *P. lambertiana* was negative and steadily decreased from 0–2 m, tracking the lower boundary of the simulation envelope, indicating spatial inhibition at these scales. From 2–4 m the large-diameter *P. lambertiana* 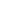 curve sharply increased, providing evidence of spatial clustering at these scales, with continued evidence for clustering occurring out to 22 m, where the empirical value reached the upper bound of the simulation envelope. Large-diameter *A. concolor* also exhibited rapid changes in spatial pattern at small scales, with steadily decreasing 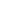 values from 0–3 m (evidence of spatial inhibition) then increasing sharply from 3–5.5 m, indicating strong spatial clustering over this short range of scales. A sustained increase in the 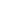 curve for large-diameter *A. concolor* from 13–38 m provided further evidence for spatial aggregation at larger scales. The 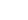 curves for large-diameter *C. decurrens* stems rose steadily from 0–80 m, reaching the upper bound of the simulation envelope at 30 m, indicating moderate but consistent clustering across these scales.

The relative spatial patterns of large- and small-diameter trees differed for all species combined, as well as for *P. lambertiana* and *A. concolor*, but not for *C. decurrens*. Small-diameter *P. lambertiana* were always more aggregated than large conspecifics at the same scale. Large-diameter *A. concolor* were less aggregated than conspecific small-diameter trees at scales of 0–3 m, then rapidly became more aggregated than small trees from 3–6 m, and remained so up to 80 m (Fig. 4). The spatial pattern of large and small *C. decurrens* subpopulations was similar from 0–80 m (Fig. 4).

**Figure 5:**
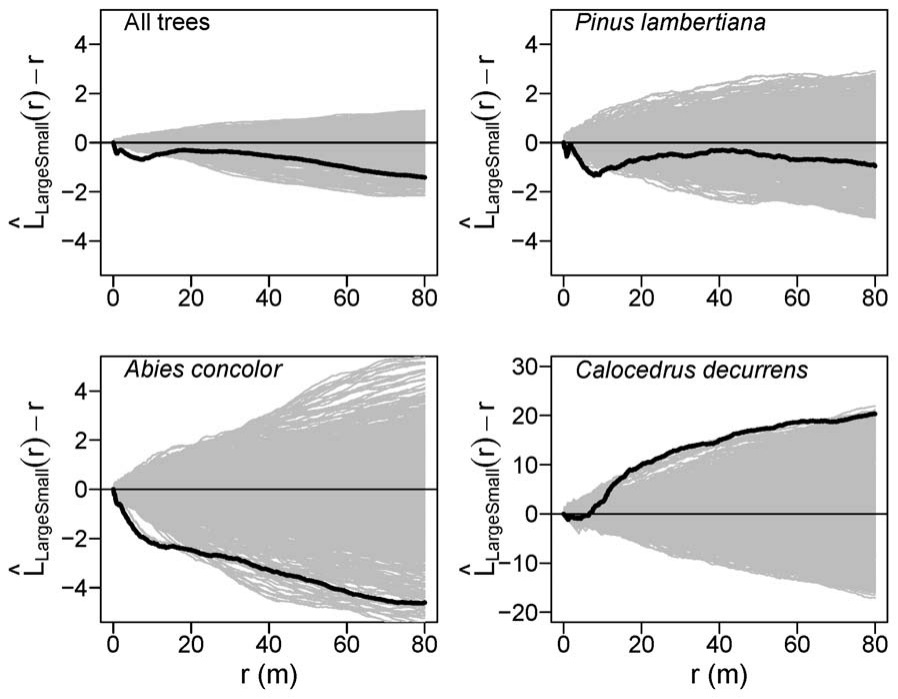
Spatial interactions between large-diameter and small-diameter trees. Solid black lines show the 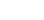 statistic for the actual pattern, where *r* is the intertree distance; thin gray lines show 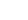 curves for 999 patterns simulated by synchronous random torodial shifts of large and small tree subpopulations. Positive values indicate spatial attraction and negative values indicate spatial repulsion. Large-diameter trees are ≥100 cm dbh; small-diameter trees are <100 cm dbh.

We found evidence for negative associations between large-diameter and small-diameter *P. lambertiana* and *A. concolor*, and for all tree species combined, relative to the population independence hypothesis when evaluated from 0–9 m (Monte Carlo goodness-of-fit tests; *A. concolor*: *P* = 0.001; *P. lambertiana*: *P* = 0.001; all trees: *P* = 0.001). Spatial locations of large-diameter and small-diameter *C. decurrens* were independent at the 9 m neighborhood scale (Monte Carlo goodness-of-fit test; *P* = 0.378). The 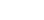 curve for *P. lambertiana* (Fig. 5) indicates spatial repulsion between large and small from 0–10 m, and modest attraction from 10–40 m. The 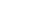 curve for *A. concolor* decreased steadily from 0–80 m, but was only outside the simulation envelope at scales less than 10 m. Large and small stems of *C. decurrens* were spatially attracted from 15–80 m, with the empirical 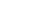 curve at or beyond the upper boundary of the simulation envelope (Fig. 5).

## Discussion

The relative proportion of large trees varies in old-growth forests worldwide [28], and at 49.4%, the contribution that large-diameter trees make to the total biomass of the YFDP is higher than in most other forests. Although some forests have almost all of their biomass concentrated in large-diameter trees (most notably *Sequoia sempervirens*; [13], [36]), the biomass of most forest types is concentrated in trees <100 cm dbh. In a 1 ha plot in tropical moist forests of Rondônia, Brazil, Brown et al. [1] found that three trees ≥100 cm dbh had biomass of 64.3 Mg compared to a total aboveground biomass of 285 Mg (22.6%). In 5.15 ha of neotropical lowland rain forest in Costa Rica, Clark & Clark [2] found that trees ≥70 cm dbh comprised 27% of the biomass of 241 Mg/ha (∼18% for trees ≥100 cm dbh; [2], Fig. 1). In semi-evergreen forests of northeast India, Baishya et al. [37] found ∼12% of biomass in trees ≥100 cm dbh, and plantation forests or forests that are recovering from disturbance may have few or no large-diameter trees, even when stem density and diversity are high [37], [38].

Within the Smithsonian Center for Tropical Forest Science network (http://www.ctfs.si.edu/plots/), only the *Gilbertiodendron dewevrei* (mbau) forest of the Congo has a higher live biomass, with the dipterocarp forests of Malaysia having equivalent live biomass (Table 4, [39], [40]). Other old-growth forest types have a biomass of ∼60% of the YFDP [39]. When the live and dead biomass are considered together, the biomass of the YFDP is 652.0 Mg/ha, currently the highest in the CTFS network. Unlike either of the high-biomass tropical plots, live biomass in the YFDP is dominated by two tree species (both Pinaceae), *Pinus lambertiana* (50.4% biomass) and *Abies concolor* (42.6% biomass), while down woody debris biomass is similarly dominated by these two species (57% and 32%, respectively). Scaling theory did not describe the distribution of biomass in this system (Fig. 2). Differences between theory and this forest are likely driven by the reoccurrence of fire throughout the period of stand development, and because of mortality rates that vary with diameter class. However, the very high levels of heterogeneity in density and biomass at 20 m scales (Fig. 1) would make scaling theory even less informative in study areas smaller than the YFDP.

**Table 4:** Comparison of the Yosemite Forest Dynamics Plot with other Smithsonian CTFS-affiliated forest plots.

Location	Latitude	Forest type	Live biomass (Mg/ha)	Live and dead biomass (Mg/ha)	Woody species	Citation
Changbaishan, China	42.2°N	Korean pine mixed forest	318.9		52	Hao et al. [41]
Yosemite, USA	37.8°N	Mixed-conifer forest	507.9	652.0	23	This study
BCI, Panama	9.2°N	Lowland tropical moist forest	306.5		299	Chave et al. [39]
Lambir, Malaysia	4.2°N	Mixed dipterocarp forest	497.2		1,182	Chave et al. [39]
Lenda, Congo	1.3°N	Mbau forest	549.7		423	Makena et al. [40]

Live biomass includes woody stems ≥1 cm dbh. Live and dead biomass includes snags ≥10 cm dbh and forest floor components as well as live biomass (also see [38] for basal area comparisons among additional large forest plots.

Although the YFDP has high biomass, the diversity of woody plants ≥1 cm dbh is the lowest among the CTFS plots ≥25 ha. The combination of summer drought and winter snow may reduce the species pool. Other temperate plots (Changbaishan, Wabikon, and the Smithsonian Ecological Research Center, SERC) have higher species diversity [41], [42]. However, those plots either receive precipitation evenly distributed throughout the year (Wabikon and SERC), or the wet season coincides with the growing season (Changbaishan).

One almost ubiquitous difficulty in biomass analyses of large-diameter trees is the uncertainty of allometric equations. The use of previously published equations to predict biomass of large trees from ground-level measurement of DBH assumes that these equations were based on adequate sampling of large trees. However, most allometric equations for tree biomass have been developed from dissection of 10–50 trees [43], and the number of large trees used in formulating equations is very low. Some of the large-diameter *P. lambertiana, A. concolor*, and *C. decurrens* exceeded the maximum diameter of any that have been dissected, and for these individual trees, substitute species were used [see Appendix S1]. Moreover, DBH is often a poor predictor of whole-tree biomass as large tree DBH is a poor reflection of tree size [10]. Nonetheless, many comparative studies of primary forest biomass use allometric equations that probably predict large-diameter tree biomass poorly (i.e. [39], [40]). Our calculations (Table 3) have been presented to a level of 1 kg/ha to enable comparison of forest dominants with less common and smaller tree species and likely represent an underestimation of whole tree biomass. However, the SD of biomass for principal species in the YFDP is 17% to 32% of the calculated value (Table 3), so the uncertainty of the large-diameter biomass could be larger than the smaller biomass pools. Biomass calculations for shrubs, snags, and woody debris also embody several simplifying assumptions (i.e. uniform stem density per unit area, single measures of diameter, simple geometry, no hollows in snags) that could lead to imprecise biomass totals.

Unlike the tropical forests where decomposition of snags and woody debris is rapid, the YFDP features considerable biomass of standing and down woody debris, also a characteristic of temperate broadleaf forests [44]. The combination of snowy winters and dry summers contributes to slow decomposition, and even the fastest decomposing tree species (*A. concolor*) has a half-life of 14 years [14]. Large-diameter snags account for a relatively high proportion of total snag biomass, while large-diameter down woody debris accounted for a lower proportion of the woody debris. This may be due to low-severity fires in the historical period that might have consumed large-diameter woody debris via glowing combustion, decreasing the proportion relative to snag representation [45], or because small snags tend to fall over relatively quickly, thus being better represented in the woody debris pool. The lack of spatial correlation of the woody debris at any scale suggests that, while mortality may be non-random, tree and snag-fall events may result in loss of spatial pattern between standing individuals and the patterns of down wood they produce.

The observed univariate spatial patterns of large and small trees provide modest support for the inference that past competition contributed to the present spatial distribution of large-diameter trees. In particular, the increasing spatial uniformity from small to large size classes is consistent with competition theory [30], [31]. However, the observed random arrangement of large trees at neighborhood scales differs from spatial uniformity expected when competition is the dominant process affecting tree spatial patterns. Previous studies of large-diameter tree spatial patterns in Sierra Nevada mixed-conifer forests agree with our findings (N.B., with large-diameter thresholds differing somewhat from 100 cm). Van Pelt and Franklin [32] found that main canopy trees at Giant Forest in Sequoia National Park were not different from spatial randomness at scales <9 m. In addition, their empirical 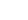 curve [32] was similar to those for *P. lambertiana* and *A. concolor* in the YFDP: inhibited from 0–1.5 m. The observed small-scale (0–3 m) inhibition is most likely due to physical requirements for minimum hard core spacing due to the large size of the boles and limits to crown plasticity, although resource competition may contribute as well. At Teakettle Experimental Forest (an old-growth, mixed-conifer forest 100 km south of the YFDP), stems ≥76 cm dbh were randomly arranged from 0–60 m [46]. However, Bonnicksen and Stone [47] found that main canopy *P. lambertiana* and *A. concolor* trees were uniformly spaced in a giant sequoia mixed-conifer forest in Kings Canyon National Park. Past competition and competitive mortality undoubtedly influenced the development of spatial patterning in large-diameter tree populations in some Sierra Nevada mixed-conifer forests [48], [49]. However, it appears that the cumulative effects of any past self-thinning in the YFDP were not sufficient to completely override the effects of clustered or random tree regeneration [50], non-random mortality or other potential sources of heterogeneity in the distribution of large-diameter trees.

We must thus consider processes other than competition to explain spatial patterns of large-diameter trees in the YFDP. For the fire-tolerant and modestly shade-tolerant *P. lambertiana*, meso-scale aggregation (2–22 m) in the large-diameter subpopulation is most readily explained by clustered establishment, consistent with a disturbance-centric model of forest dynamics and spatial pattern formation in low and mixed severity fire regimes [51]. For *A. concolor* (fire intolerant when small) the strong clustering of large-diameter trees at local (3–5.5 m) and intermediate (13–38 m) scales may originate from fire refugia that allowed groups of *A. concolor* to survive and reach large diameters. Clustered establishment alone (e.g., in gaps or in moisture-receiving microtopographic features) could explain the aggregation of large *A. concolor* stems, but given the historical regime of frequent fire [51] it is likely that heterogeneous fire effects leading to patchy *A. concolor* survival also contributed.

The observed spatial segregation of large and small trees is consistent with inference that competitive interactions between these size classes influence their spatial relationships and overall forest structure. Spatial segregation of large and small trees has been documented in many other forest types (i.e. [33] and the studies reviewed therein), including Sierra Nevada mixed-conifer forests [32]. Spatial segregation between large and small trees may arise from asymmetrical competition for light and gap-phase regeneration [33]. However, we acknowledge that other mechanisms acting at the tree neighborhood scale potentially contribute to the observed spatial segregation between large and small *A. concolor* and *P. lambertiana*, including crushing mortality by falling limbs and bole fragments from live large-diameter trees [8], [52], and the spatially heterogeneous buildup and subsequent burning of surface fuels. Additionally, in the absence of fire large-diameter *P. lambertiana* accumulate a deep mound of debris (bark and needles) at their base [53], a substrate not suitable for seedling establishment, which would also give rise to repulsion between large and small stems. Prior to fire exclusion, Sierra Nevada mixed-conifer forests had low densities of small-diameter trees [35], [51]; the observed repulsion between tree diameter classes may also be due to preferential tree establishment in fire-maintained openings following disruption of the historical fire regime [54].

### Conclusions

We assessed the degree to which scaling theory and competition theory explain variation of accumulated biomass and spatial patterns across the tree size spectrum. These respective bodies of theory were not sufficient to explain our empirical results. However, our results do not indicate the rejection of these theories. Scaling theory is clearly a powerful framework for developing novel ecological insights, but our results and those of others [21], [27], [28] show that the requisite simplifying assumptions render predictions from scaling theory inappropriate as inputs in to ecosystem models or as a basis for natural resource decision making. A vast body of accumulated scientific literature details mechanisms and outcomes of plant competition; our results do not contradict this theory. Rather, competition theory alone was insufficient to explain our empirical measurements of tree spatial patterns, strengthening the conclusion that competition is not the dominant control of tree population dynamics and forest development in old-growth Sierra Nevada mixed-conifer forests [24].

We predict that long-term observations at our study site and other sites throughout the range of Sierra Nevada mixed-conifer forests will reveal strong top-down regulation of forest biomass and spatial structure by pathogens, insects and physical disturbances, especially in old-growth forests. We also suggest that, in forests with high functional inequality across the tree size spectrum, ecosystem function may be more sensitive to natural perturbations, environmental change or management actions – at least those affecting the large-diameter trees – than in forests where ecosystem function is distributed more equitably across the tree size spectrum. Sustaining ecological functions and services, such as carbon storage or provision of habitat for specialist species, will likely require different forest management strategies when the ecosystem services are provided primarily by a few large trees as opposed to many smaller trees.

## Materials and Methods

### Study area

**Figure 6:**
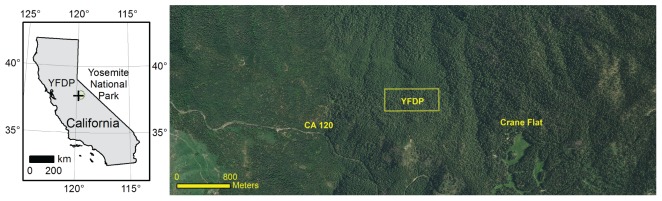
Location of the Yosemite Forest Dynamics Plot (YFDP). The YFDP is located near the western boundary of Yosemite National Park (left, green) in the lower montane, mixed-conifer zone of the Sierra Nevada, California, USA. The plot is located in relatively uniform, late-successional forest near Crane Flat (right). The area immediately north of the YFDP was logged in the early 1930s, as was the area comprising the western 1/3 of the image.

**Figure 7:**
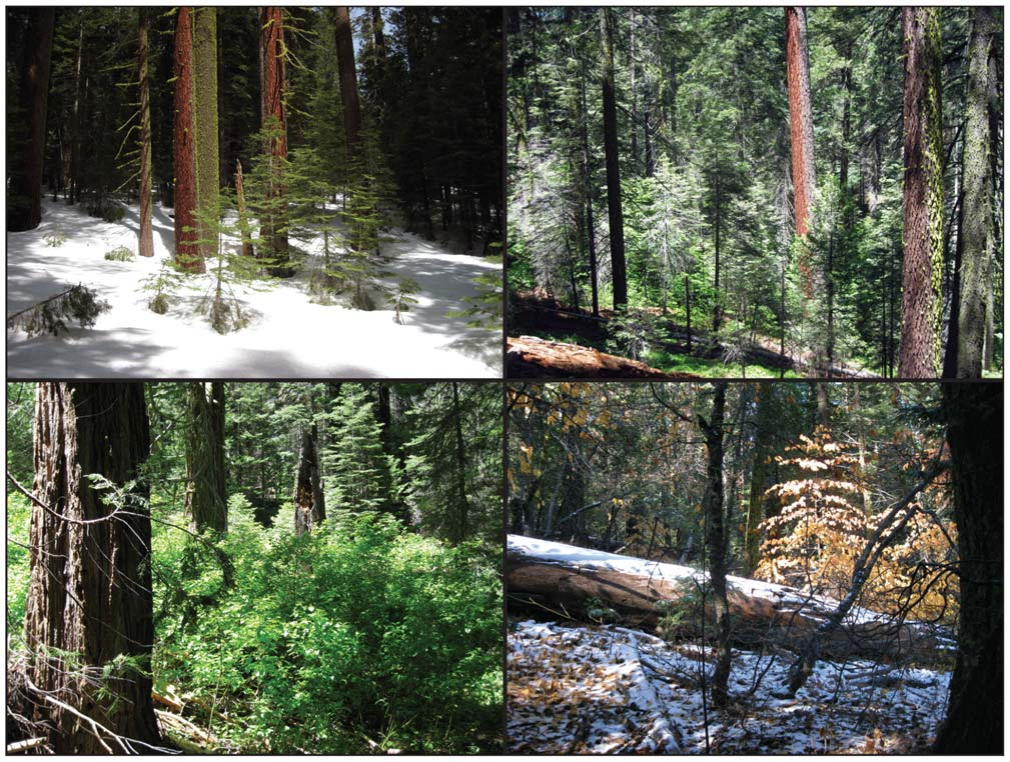
Structure and composition of the Yosemite Forest Dynamics Plot (YFDP). Four images from different parts of the YFDP illustrate defining characteristics of the ecosystem. Most precipitation falls in the winter as snow yielding a spring snowpack of approximately 1 m (upper left image, April 11, 2011). The forest is composed of an overstory of large-diameter trees with abundant but heterogeneous shrub and herbaceous layers (upper right image, June 22, 2009). Shrubs can be locally dense enough to reduce tree recruitment (lower left image, June 22, 2009). Although most trees and shrubs are evergreen, the presence of the deciduous species *Cornus nuttallii* and *Quercus kelloggii* results in seasonal openings in the canopy (lower right image, November 11, 2010). All photos by J. A. Lutz.

The Yosemite Forest Dynamics Plot (YFDP) is located in the mixed-conifer forest of the western portion of Yosemite National Park (Fig. 6). The plot is approximately oriented to the cardinal directions with dimensions of 800 m east to west and 320 m north to south (25.6 ha) centered at 37.77°N, 119.82°W. Elevation ranges between 1774.1 m and 1911.3 m for a vertical relief of 137.2 m (Fig. S1). The YFDP is comprised of vegetation types within the *Abies concolor – Pinus lambertiana* Forest Alliance [55], including *Abies concolor-Pinus lambertiana/Ceanothus cordulatus* Forest, *Abies concolor-Pinus lambertiana/Maianthemum racemosum* (*Smilacina racemosa*, Hickman [56])-*Disporum hookeri* Forest, *Abies concolor-Calocedrus decurrens-Pinus lambertiana/Cornus nuttallii/Corylus cornuta* var. *californica* Forest, *Abies concolor-Calocedrus decurrens-Pinus lambertiana/Adenocaulon bicolor* Forest, and *Abies concolor-Pinus lambertiana- Calocedrus decurrens/Chrysolepis sempervirens* Forest, classified according to the U.S. National Vegetation Classification [57](Fig. 7). Overall demographic rates in Sierra Nevada conifer forests between 1500 m and 2000 m elevation are approximately 1.5% [58], [59]. Canopy emergents, principally *P. lambertiana* and *A. concolor*, reach 60 m to 67 m in height. The soils of the YFDP are derived from metamorphic parent material. Approximately 85% of the soils of the YFDP are metasedimentary soils of the Clarkslodge-Ultic Palexeralfs complex with a water-holding capacity of 160 mm in the top 150 cm of the soil profile [60]. The soils of the northwest 15% of the YFDP are Humic Dystroxerepts-Typic Haploxerults-Inceptic soils of the Haploxeralfs complex with a water-holding capacity of 70 mm in the top 150 cm of the soil profile [60]. Plant nomenclature follows Hickman [56].

The climate at the YFDP is Mediterranean, with cool moist winters and long dry summers. Between 1971 and 2000, the modeled mean temperature range at the YFDP was from 12.2°C to 26.1°C in July and −2.7°C to 9.4°C in February; annual precipitation was 1061 mm, with most precipitation falling in the winter months as snow [61], [62]. Snow depth on April 1^st^ is generally 100 cm to 150 cm. The seasonality of precipitation yields a summer drought with a mean annual climatic water deficit of 200 mm [63] (Fig. S2).

### Disturbance processes: fire, wind, insects, pathogens, vertebrates, and human use

Fire is the dominant natural disturbance process in Sierra Nevada mixed-conifer forests [64]. The fire regime is of mixed severity with fires burning in a mosaic of high, moderate, and low severities. The pre-Euro-American fire return interval for the YFDP was 10–13 years [51]. The combination of repeated fire and other disturbances gives rise to a fine-grained mosaic structure [50], [65]. During the Landsat TM period of record (1984–2011), most fires in this forest type have been either low severity management-ignited prescribed fires or moderate and high severity wildfires [66]–[68]. The YFDP has not burned since comprehensive park fire records were initiated in 1930. Mechanical damage, whether from wind, snow, or crushing of smaller individuals by falling trees or tree parts contributes to stand structural development [8], [69]. Many of the larger trees in the YFDP have broken tops, or reiterated tops that have regrown following damage.

Insects are important agents of mortality, with most common conifer tree species having coevolved bark beetles (family Scolytidae) that are always present at low levels [70], [71]. In particular, *Dendroctonous ponderosa* (mountain pine beetle) attacks *Pinus lambertiana* and *P. ponderosa* and *Scolytus ventralis* attacks *Abies concolor* and *A. magnifica*. Other bark beetles such as *S. subscaber, D. valens* (red turpentine beetle) and *Ips* spp. have been less abundant in the recent past but contribute to tree mortality. *Quercus kelloggii* (California black oak) also has associated bark beetles (*Pseudopityophthorus* spp.). *Conophthorus ponderosae* (ponderosa pine cone beetle) is present and can reduce the reproductive output of *P. lambertiana*.

Pathogens include the structural root rots *Armillaria* spp. [72], *Heterobasidion annosum* [73] and *Phaeolus schweinitzii* [71]. The root rots spread through roots and root contacts at rates of approximately 30 cm per year, and hence tend to occur in patches. *Armillaria* spp. are somewhat generalist pathogens and attack *Abies* spp., *Prunus* spp., and *Cornus nuttallii*. *Phaeolus schweinitzii* infects *Pinus lambertiana* and *Abies* spp., but tends to progress much more slowly than *Heterobasidion* and *Armillaria*. *Pinus lambertiana* is also affected by the introduced pathogen *Cronartium ribicola* [74]. *Calocedrus decurrens, Abies concolor*, and *Quercus kelloggii* are hosts to mistletoes: *Phoradendron libocedri* on *C. decurrens*, *Phoradendron pauciflorum* and *Arceuthobium abietinum* on *A. concolor*, and *Phoradendron villosum* on *Q. kelloggii* [75]. These mistletoes are distributed both by birds, and in the case of *Arceuthobium abietinum*, also by explosive discharge that can carry seeds up to 16 m (typically 10 m; [75]).

The YFDP has a rich fauna, with most species of herbivores and their predators present since prior to Euro-American settlement. Large mammals include *Ursus americanus* (black bear), *Felis concolor* (mountain lion), *Canis latrans* (coyote), and *Odocoileus hemionus* (mule deer). Altogether, vertebrate species observed within similar forest types within 5 km of the YFDP include 16 rodent species, 12 bat species, 7 carnivore species, one hooved mammal, 7 raptor species, 38 passerine species, 5 amphibian species, and 7 reptile species Table S1, [76]–[79]).

Yosemite has been inhabited at least since 100 AD [80]. Immediately prior to Euro-American discovery of the region in 1833 [81] and the subsequent entry of Euro-Americans into Yosemite Valley in 1851 [82], the area was occupied by the Central Sierra Miwok and the Southern Sierra Miwok [83]. Because the YFDP contains only intermittent streams and seeps, Native American use of the site was probably low, and modification of the fire regime at this site by Native Americans appears unlikely [84]. The YFDP is near the transit route from Hazel Green to Crane Flat used by sheepherders in the late 19^th^ century. John Muir may have passed through or near the YFDP on July 9^th^, 1869 – the topography and vegetation are consistent with his journal entry [85].

The original Yosemite Grant (1864) placed Yosemite Valley and the Mariposa Grove of giant sequoia in protected status. The YFDP lies within what was a single parcel of land prior to its inclusion into Yosemite National Park in 1930. The parcel of land immediately to the north of the YFDP (∼20 m from the plot boundary) was in different ownership and was logged in the early 1930s. The northwest corner of the YFDP contains four large stumps that appear to be associated with the logging of the parcel to the north. Logging outside the YFDP continued throughout the 1920s until the area was purchased by the National Park Service and John D. Rockefeller. The area of unlogged sugar pine containing the YFDP is today termed the Rockefeller Grove in honor of J.D. Rockefeller's role in protecting this part of the park.

### Surveying

We established a sampling grid using Total Stations with accuracies of at least 5 seconds of arc (Leica models 1100, Builder R200M Power, Builder 505, and TC 2003). We set permanent markers on nominal 20 m centers, offset for tree boles, coarse woody debris, or large rocks. Survey closure across the plot was 0.18 m northing, 0.05 m easting, and 0.03 m elevation (∼1/5000). In addition to the sampling grid, we established control points in open areas near the plot where marginal Global Positioning System (GPS) reception was possible. Three survey-grade GPS receivers (Magellan Z-Extremes) were used to establish control to and across the plot, using a reference station approximately 2 km from the plot (MGROVE, PID DF8617 on the California State Plane Coordinate System and being described in the National Geodetic Survey Datasheets). The GPS receivers collected data at 10 second intervals for 2–6 hours. The static GPS measurements were post-processed with GNSS Solutions software (Magellan Navigation, Inc., pro.magellangps.com), with final accuracies in the range of 0.01 m horizontally and 0.02 m vertically. We transformed the plot grid to Universal Transverse Mercator coordinates with Lewis and Lewis Coordinated Geometry software (Lewis and Lewis Land Surveying Equipment, Inc., www.lewis-lewis.net). We augmented the ground survey with LiDAR-derived elevation data at 1 m horizontal resolution. Aerial LiDAR data were acquired on 22 July 2010 by Watershed Sciences Inc., Corvallis, Oregon with a density of 40 returns per square meter. Ground survey data and the LiDAR-derived ground model coincided with a root-mean-squared error of 0.15 m.

### Field sampling of trees, shrubs, snags, and woody debris

In the summers of 2009 and 2010 we tagged and mapped all live trees ≥1 cm at breast height (1.37 m; dbh), following the methods of Condit [86], with some alterations. We measured tree diameter at 1.37 m (instead of 1.30 m), and trees large enough to accept a nail were nailed at the point of measurement, both in keeping with research methods of the western United States. We used stainless steel tags, nails and wire to increase tag longevity in this fire-dominated ecosystem. We measured tree locations from the surveyed grid points with a combination of hand-held lasers (Laser Technologies Impulse 200 LR), mirror compasses, and tapes. Tapes were laid south to north between adjacent grid points, and a perpendicular angle determined by sighting a target bole with a mirror compass. The distance from the tape to each tree was then measured with the hand-held lasers. We calculated the location of the tree center from the horizontal and perpendicular references to the surveyed grid points and dbh with the assumptions of cylindrical boles and linear interpolation of elevation between adjacent grid points. All measurements were slope corrected.

We mapped continuous patches of shrub cover ≥2 m^2^ relative to the 20 m sampling grid with a combination of tapes, mirror compasses, and lasers. For each shrub patch we recorded the shape of the patch as a polygon, as well as average and maximum shrub heights. To convert between shrub cover and the number of stems and biomass in the YFDP, we established 25 shrub demography plots for nine species (*Arctostaphylos patula, Ceanothus cordulatus*, *Ceanothus integerrimus, Ceanothus parvifolius*, *Chrysolepis sempervirens, Corylus cornuta* var. *californica*, *Cornus sericia, Leucothoe davisiae*, and *Vaccinium uliginosum*). We tagged every woody stem in each of these 2 m×2 m plots. We measured basal diameter for every woody stem. If stems were 1.37 m tall (or long), we made an additional measurement at 1.37 m.

We tagged and mapped dead trees ≥10 cm dbh and ≥1.8 m in height. For each snag, we collected height, top diameter (with a laser), and snag decomposition class data (following [87]; class 1 = least decayed, class 5 = most decayed). We did not collect data on trees <10 cm dbh at the original census because of the difficulty in finding small stems a few years after they die.

To measure down woody debris, litter, and duff, we established four interior fuel transects totaling 2.24 km (112 transects of 20 m). We used the National Park Service fuel monitoring protocols [88], in turn based on Brown transects [89]. Litter included freshly fallen leaves, needles, bark, flakes, acorns, cones, cone scales, and miscellaneous vegetative parts [88]. Duff included the fermentation and humus layers, not the fresh material of the litter layer. Down woody material included branches, trunks of trees, and shrubs that had fallen on or within 2 m above the ground [88]. Intercept diameter and decay class were recorded for all intercepted woody debris ≥10 cm in intercept diameter (measured perpendicular to the orientation of the piece of debris). To sample fine woody debris we used portions of the 112 line intercept transects −2 m for material 0 cm–2.5 cm in diameter (1-hour and 10-hour fuels), and 4 m for material 2.5 cm–7.6 cm (100-hour fuels). We calculated biomass according to Brown's method [89].

### Biomass calculations

We reviewed all allometric equations from the two compendia of equations for North America [43], [90] and selected those that best matched the species, geographic location, diameter ranges, and tree densities of the YFDP [44], [90]–[93]. Where no allometric equation existed, we substituted a species (or diameter class within a species) that was a close match for morphology and wood density (see Appendix S1 for details). Because no whole tree biomass equations exist for the largest individuals of the species in the YFDP, we used proxy species. For the largest *Abies concolor* (n = 112) we used bole equations for *A. procera*. For *Pinus lambertiana*, we used branch and foliage equations for *Pseudotsuga menziesii*, and for the *Pinus lambertiana* >179.6 cm dbh (n = 7), we used a bole equation for *Pseudotsuga menziesii*. Additionally, no biomass equations exist for branches and foliage of *Abies* >110 cm dbh or *Pseudotsuga* >162 cm dbh. For those trees we capped the branch and foliage biomass at the values associated with trees of diameter 110 cm and 162 cm, respectively. All biomass calculations were made within the data ranges of the selected allometric equations (See Appendix S1 for full details of allometric equations). We calculated an error term for tree biomass from the underlying allometric equations. The root mean square error (standard error of estimate) of the allometric equations was transformed from log units to arithmetic units of standard deviation (i.e. Mg/ha) [92]. We defined large diameter structures as pieces ≥100 cm in diameter to facilitate comparisons with earlier studies of large-diameter trees in old-growth conifer forests on the Pacific Slope of western North America.

We calculated the biomass of the stems within each 4 m^2^ shrub demography plot based on allometric equations using basal diameter [90]. We used the biomass of the stems within the demography plots and the total area of shrub patches ≥2 m^2^ within the YFDP to calculate total shrub biomass. We used the demography plot data for sampled species as proxies for the four species without demography plots (*Corylus cornuta* var. *californica* for *Sambucus racemosa, Vaccinium uliginosum* for *Rhododendron occidentale* and *Ribes nevadense*, and one-half the value of *Vaccinium uliginosum* for *Ribes roezlii*). To calculate a stem density equivalent to the standard Smithsonian CTFS protocol [86], we tallied the number of stems that were ≥1 cm dbh in each shrub demography plot and multiplied by the area of each shrub patch ≥2 m^2^. Details of allometric equations are in Appendix S1.

We calculated snag biomass using the wood density values of Harmon et al. [15] and a bole volume calculated as a frustum of a cone. We calculated the biomass of litter and duff using the methods of Stephens et al. [94]. For down woody debris larger than 1000-hour fuels (4 inches; ∼10 cm), we used the large transect protocols of Harmon et al. [15], and we calculated the mass of woody debris using the methods of Harmon and Sexton [87].

To compare actual density and biomass values with the predictions of scaling theory, we used the equations from West et al. [26]. Specifically, we compared the actual diameter (radius) distribution with their predicted distribution, 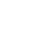, where *r* is tree radius at breast height. We then reconfigured their radius-mass relationship, 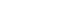, to 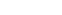, where *r* is tree radius and *m* is tree biomass, and combined the mass and frequency equations to develop a relationship for total biomass in terms of tree radius: 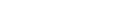 or 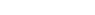. We used 5 cm diameter bins (2.5 cm radius bins) to regress curves of these forms to the data.

### Quantifying spatial pattern

We quantified global spatial patterns with the univariate and bivariate forms of Ripley's *K* function, using the square root (*L* function) transformation in all cases. For a given fully mapped pattern, an estimate of the *L(r)* function, the statistic 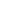, is based on the count of neighboring points occurring within a circle of radius *r* centered on the *i*th point, summed over all points in the pattern [95], [96]. The bivariate form 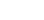 is a straightforward extension of the univariate case: it is the count of type 2 points occurring within a circle of radius *r* of the *i*th type 1 point, summed over all type 1 points in the pattern. We characterized patterns at interpoint distances from 0 m to 80 m (one quarter the minimum plot dimension) and used isotropic edge correction to account for points located closer than *r* to a plot edge [96]. Our study area included enough large-diameter trees to analyze spatial patterns of three tree species: *Abies concolor*, *Calocedrus decurrens* and *Pinus lambertiana*.

### Inferential framework for spatial analyses

Univariate tree patterns were compared against a null distribution generated by a completely spatially random (CSR) process. Under CSR the location of each point in the pattern is completely independent of the locations of other points in the pattern. Positive values of 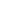 indicate spatial clustering (trees have more neighbors than expected under CSR) while negative values of 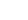 indicate spatial inhibition or uniformity (trees have fewer neighbors than expected under CSR).

Bivariate tree patterns were evaluated against the hypothesis of no interaction between the large-diameter and small-diameter subpopulations. We evaluated this hypothesis using the null model of population independence based on the guidelines of Goreaud & Pélissier [97]. Population independence is evaluated by holding the relative intratype spatial configuration constant (i.e., the relative tree locations within a diameter class are fixed) while subjecting the populations to random toroidal shifts. Under population independence significantly positive values of 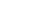 indicate a spatial attraction between the two types (e.g., originating from a parent-offspring relationship or facilitation) while significantly negative values indicate spatial repulsion between the two types (e.g., Janzen-Connell effects or intraspecific competition). Large-diameter trees were ≥100 cm dbh; small-diameter trees were <100 cm dbh.

We used the 9 m radius neighborhood size estimated by Das et al. [24], [98] for Sierra Nevada mixed-conifer forests and tested the respective empirical patterns against the corresponding null models over 0 m≤*r*≤9 m using the goodness-of-fit test developed by Loosmore and Ford [99]. We set α = 0.05 and used *n* = 999 simulated patterns in each test (*n* = 250 simulated patterns were used for univariate analyses of small-diameter *A. concolor* and all species pooled, respectively, to mitigate excessively long computation times). To control for multiple tests (*n* = 12) we used the Bonferroni correction, resulting in a threshold *P*-value of 0.004. Because we had no *a priori* hypotheses about tree patterns at spatial scales >9 m we investigated patterns at larger scales in an exploratory framework by comparing the empirical 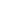 curves to the full distribution of 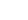 curves calculated for the simulated patterns. All analyses were implemented in the statistical program R version 2.14.1 [100]. Spatial analyses were conducted using the spatstat package version 1.25-1 [101].

## Supporting Information

Figure S1 **Topography of the Yosemite Forest Dynamics Plot.** LiDAR-derived ground model at 1 m resolution (5 m contours; 137.2 m vertical relief). Dots indicate corners of each 20 m×20 m quadrat of the 800 m×320 m plot. Elevation ranges from 1774.1 m in the northeast corner to 1911.3 m along the southern boundary for a vertical relief of 137.2 m. Drainages contain vernal streams.
(TIF)

Figure S2 **Climatology and water balance of the Yosemite Forest Dynamics Plot.** The combination of temperature and precipitation (A) give rise to a pronounced summer drought (B). Potential evapotranspiration (PET) exceeds available water supply from May through September, decreasing actual evapotranspiration (AET) and producing a climatic water deficit (Deficit) of 197 mm of water.
(TIF)

Table S1 **Vertebrate species reported in similar forest types within 5 km of the Yosemite Forest Dynamics Plot between 1980 and 2011.**
(PDF)

Appendix S1 **Allometric equations for total aboveground biomass for trees ≥1 cm dbh and shrubs in patches of continuous cover ≥2 m^2^ in the Yosemite Forest Dynamics Plot.**
(PDF)
