# Supplementary figures and images for: Ecological Importance of Large-Diameter Trees in a Temperate Mixed-Conifer Forest
Source: PLoS One. 2012 May 2;7(5):e36131. doi: 10.1371/journal.pone.0036131 (PMC3342248; doi:10.1371/journal.pone.0036131)

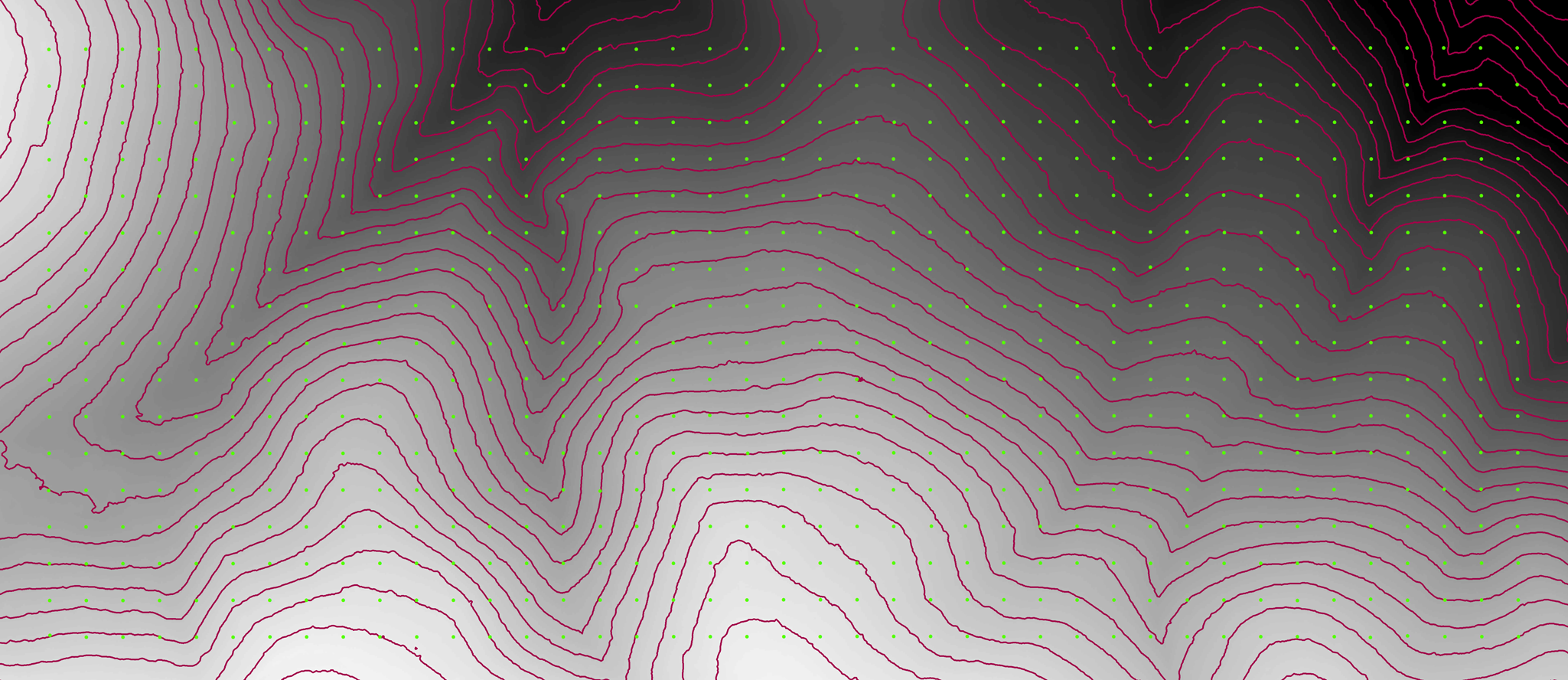

Supplement: Figure S1 — Topography of the Yosemite Forest Dynamics Plot. LiDAR-derived ground model at 1 m resolution (5 m contours; 137.2 m vertical relief). Dots indicate corners of each 20 m×20 m quadrat of the 800 m×320 m plot. Elevation ranges from 1774.1 m in the northeast corner to 1911.3 m along the southern boundary for a vertical relief of 137.2 m. Drainages contain vernal streams. (TIF) [file pone.0036131.s001.tif]

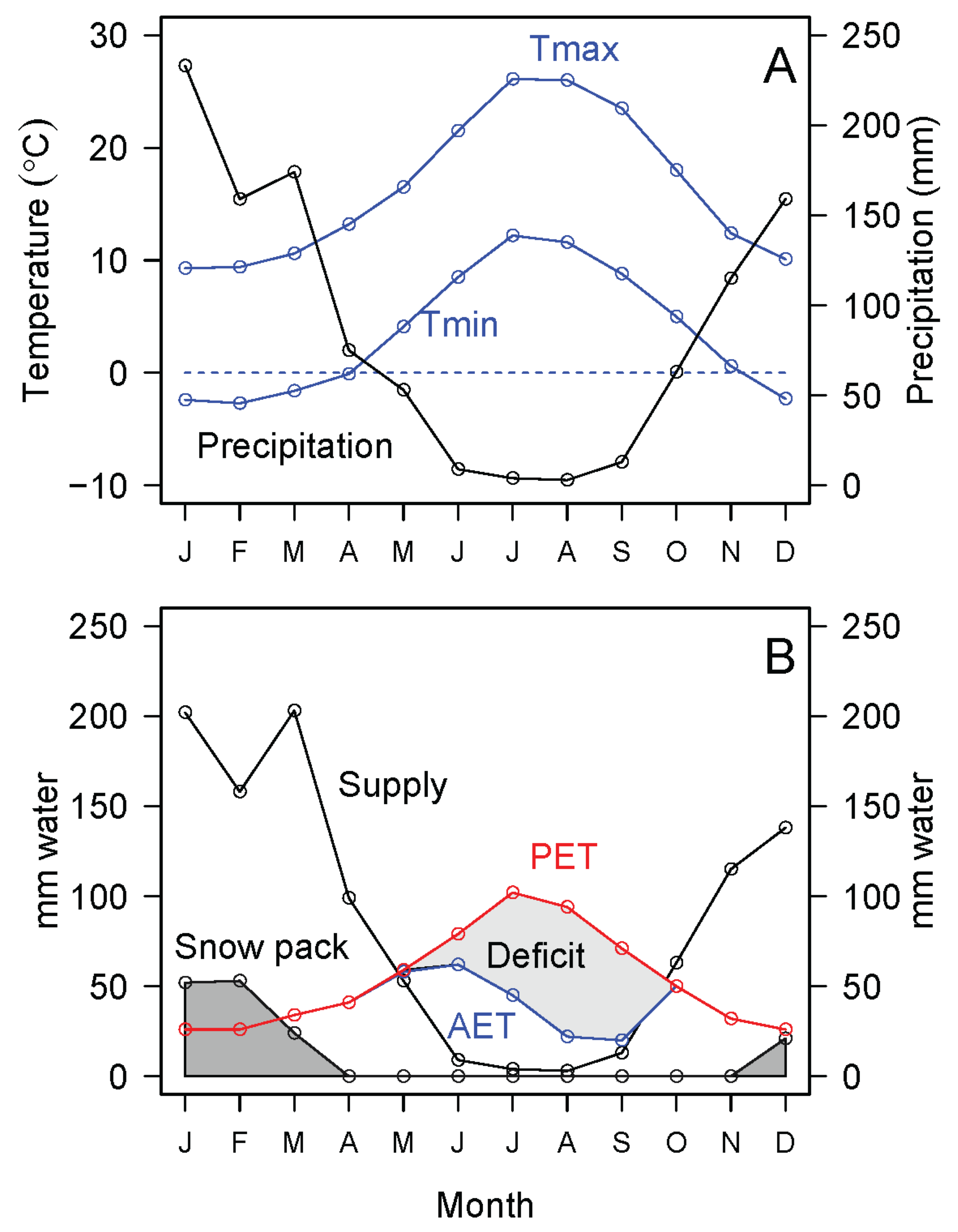

Supplement: Figure S2 — Climatology and water balance of the Yosemite Forest Dynamics Plot. The combination of temperature and precipitation (A) give rise to a pronounced summer drought (B). Potential evapotranspiration (PET) exceeds available water supply from May through September, decreasing actual evapotranspiration (AET) and producing a climatic water deficit (Deficit) of 197 mm of water. (TIF) [file pone.0036131.s002.tif]
