# Supplementary material for: Ecological Importance of Large-Diameter Trees in a Temperate Mixed-Conifer Forest
Source: PLoS One. 2012 May 2;7(5):e36131. doi: 10.1371/journal.pone.0036131 (PMC3342248; doi:10.1371/journal.pone.0036131)
Supplement: Table S1 — Vertebrate species reported in similar forest types within 5 km of the Yosemite Forest Dynamics Plot between 1980 and 2011. (PDF) [file pone.0036131.s003.pdf]

Table S1 Vertebrate species reported in similar forest types within 5 km of the Yosemite Forest Dynamics Plot between 1980 and 2011. Data compiled from [S1.1-S1.5].

Scientific name	Common name	Data source
MAMMAL SPECIES		
Rodent species		
Glacomys sabrinus	northern flying squirrel	LiveTrap
Microtus longicaudus	long-tailed vole	LiveTrap
Neotamias quadrimaculatus	long-eared chipmunk	LiveTrap
Neotamias senex	shadow chipmunk	LiveTrap
Neotoma spp	woodrat (probably bushy-tailed)	OwlPellet
Peromyscus boleyii	brush mouse	LiveTrap
Permomyscus maniculatus	deer mouse	LiveTrap
Peromyscus truei	pinon mouse	LiveTrap
Scapanus latimanus	broad-footed mole	OwlPellet
Sciurus griseus	western grey squirrel	LiveTrap
Sorex unknown species	unknown shrew	LiveTrap
Spermophilus lateralis	golden-manteled ground squirrel	LiveTrap
Spermophilus beecheyi	California ground squirrel	LiveTrap
Tamiasciurus douglasii	Douglas squirrel	LiveTrap
Thomomys monticola	mountain pocket gopher	LiveTrap
Zapus princeps	western jumping mouse	LiveTrap
Bat species		
Antrozous pallidus	pallid bat	OwlPellet
Eptesicus fuscus	big brown bat	Pierson et al. 2006
Euderma maculatum	spotted bat	Pierson et al. 2006
Eumops perotis	western mastiff bat	Pierson et al. 2006
Lasionycteris noctivagans	silver-haired bat	Pierson et al. 2006
Lasiurus cinereus	hoary bat	Pierson et al. 2006
Myotis californicus	California myotis	Pierson et al. 2006
Myotis evotis	long-eared myotis	Pierson et al. 2006
Myotis thysanodes	fringed myotis	Pierson et al. 2006
Myotis volans	long-legged myotis	Pierson et al. 2006
Myotis yumanesis	Yuma myotis	Pierson et al. 2006
Tadarida brasiliensis	Mexican free-tailed bat	Pierson et al. 2006
Carnivore species		
Urocyon cinereoargenteus	western grey fox	Observation
Felis concolor	mountain lion	Observation
Canis latrans	coyote	Observation
Ursus americanus	black bear	Observation
Mephitis mephitis	striped skunk	Theoretically present[†]

Lynx rufus	bobcat	Theoretically present†
Procyon lotor	raccoon	Theoretically present†
Hooved mammal species		
Odocoileus hemionus	mule deer	Observation
AVIAN SPECIES		
Raptor species		
Accipiter gentilis	northern goshawk	Observation
Accipiter striatus	sharp-shinned hawk	Theoretically present†
Aegolius acadicus	northern saw-whet owl	Observation
Otus flammeolus	flammulated owl	Observation
Otus kennicottii	western screech owl	Observation
Glaucidium gnoma	northern pygmy-owl	Observation
Strix occidentalis occidentalis	Calfornia spotted owl	Observation
Passerine species		
Patagioenas fasciata	band-tailed pigeon	
Piranga ludoviciana	western tanager	Observation
Turdus migratorius	American robin	Observation
Catharus guttatus	hermit thrush	Theoretically present†
Dryocopus pileatus	Pileated woodpecker	Observation
Contopus sordidulus	western wood-pewee	Observation
Contopus cooperi	olive-sided flycatcher	Theoretically present†
Empidonax hammondii	Hammond's flycatcher	Observation
Empidonax oberholseri	dusky flycatcher	Theoretically present†
Corvus corax	common raven	Observation
Cyanocitta stelleri	Stellar's jay	Observation
Colaptes auratus	northern flicker	Observation
Sphyrapicus thyroideus	Williamson's sapsucker	Theoretically present†
Sphyrapicus nuchalis	red-naped sapsucker	Theoretically present†
Sphyrapicus ruber	red-breasted sapsucker	Theoretically present†
Picoides villosus	hairy woodpecker	Theoretically present†
Picoides albolarvatus	white-headed woodpecker	Theoretically present†
Vireo cassinii	Cassin's vireo	Theoretically present†
Vireo gilvus	warbling vireo	Theoretically present†
Chaetura vauxi	Vaux's swift	Theoretically present†
Calypte anna	Anna's hummingbird	Theoretically present†
Poecile gambeli	mountain chickadee	Observation
Sitta canadensis	red-breatsed nuthatch	Observation
Sitta pygmaea	pygmy nuthatch	Theoretically present†
Certhia americana	brown creeper	Observation
Troglodytes hiemalis	winter wren	Observation

Regulus satrapa	golden-crowned kinglet	Theoretically present†
Dendroica coronata	yellow-rumped warbler	Theoretically present†
Junco hyemalis	dark-eyed junco	Observation
Oreothlypis celata	orange-crowned warbler	Theoretically present†
Bombycilla cedrorum	cedar waxwing	Theoretically present†
Oreothlypis ruficapilla	Nashville warbler	Theoretically present†
Dendroica nigrescens	black-throated gray warbler	Theoretically present†
Dendroica townsendi	Townsend's warbler	Observation
Dendroica occidentalis	hermit warbler	Theoretically present†
Loxia curvirostra	red crossbill	Theoretically present†
Spinus pinus	pine siskin	Theoretically present†
Coccothraustes vespertinus	evening grosbeak	Theoretically present†
AMPHIBIAN SPECIES		
Batrachoseps spp	slender salamander	Theoretically present†
Bufo boreas halophilus	western toad	Observation
Ensatina eschscholtzii platensis	Sierra Nevada ensatina	Observation
Pseudacris regilla	Pacific tree frog	Observation
Taricha torosa sierrae	Sierra newt	Theoretically present†
REPTILE SPECIES		
Charina bottae	northern rubber boa	Theoretically present†
Contia tenuis	sharp-tailed snake	Theoretically present†
Diadophis punctatus pulchellus	coral-bellied ring-necked snake	Theoretically present†
Elgaria coerulea palmeri	Sierra alligator lizard	Theoretically present†
Eumeces gilberti gilberti	Gilbert's skink	Theoretically present†
Sceloporus occidentalis taylori	Sierra fence lizard	Theoretically present†
Thamnophis couchii	Sierra garter snake	Theoretically present†

†Theoretically present based on http://www.nps.gov/yose/naturescience/wildlifespecies.htm as accessed on 26 August 2011

References

S1.1 Meyer MD, North MP, Kelt DA (2007) Nest trees of northern flying squirrel in Yosemite National Park, California. Southwestern Naturalist 52: 157-161.

S1.2 Pierson ED, Rainey WE, Chow LS (2006) Bat use of the giant sequoia groves in Yosemite National Park. Yosemite Fund Report, Yosemite National, California.

S1.3 Roberts SL (2008) The effect of fire on California spotted owls and their mammalian prey in the central Sierra Nevada, California. Dissertation. University of California Davis.

S1.4 Roberts SL, van Wagtendonk JW, Kelt DA, Miles AK, Lutz JA (2008) Modeling the effects of fire severity and spatial complexity on small mammals in Yosemite National Park, California. Fire Ecology 4(2): 83-104.

S1.5 Roberts SL, van Wagtendonk JW, Miles AK, Kelt DA (2011) Effects of fire on California spotted owl occupancy in a late-successional forest. Biological Conservation 144: 610-619.
