# Supplementary material for: Ecological Importance of Large-Diameter Trees in a Temperate Mixed-Conifer Forest
Source: PLoS One. 2012 May 2;7(5):e36131. doi: 10.1371/journal.pone.0036131 (PMC3342248; doi:10.1371/journal.pone.0036131)
Supplement: Appendix S1 — Allometric equations for total aboveground biomass for trees ≥1 cm dbh and shrubs in patches of continuous cover ≥2 m2 in the Yosemite Forest Dynamics Plot. (PDF) [file pone.0036131.s004.pdf]

Appendix S1 Allometric equations for total aboveground biomass for trees ≥1 cm dbh and shrubs in patches of continuous cover ≥ 2 m^2 in the Yosemite Forest Dynamics Plot.

Tree Species

We used combinations of equations to cover the species mixture and diameter range in the YFDP [S1.1-S1.4]. For some larger diameter trees, bole equations were available for the full diameter range in the data, but canopy biomass equations were not. In that case, branch and foliage biomass was capped at the largest applicable diameter for the allometric equation. For trees with diameters smaller than applicable species-specific equations (generally those with very low biomass as calculated by any choice of equation), we used two generalist proxy species: for conifers, *Pseudotsuga menziesii* [S1.5], and for angiosperms, *Alnus rubra* [S1.3].

Abies concolor (n = 24,481) – For *Abies concolor* 1.0 cm ≤ dbh < 7.0 cm dbh (n = 11,519), we used the generic small conifer proxy [S1.5]. For *Abies concolor* 7.0 cm ≤ dbh ≤ 98.0 cm (n = 12,850) we used the total aboveground biomass equation from [S1.2], representing trees sampled directly south of Yosemite National Park. For *Abies concolor* 98.0 cm < dbh ≤ 164.9 cm (n = 112), we used the bole equation for *Abies procera* from [S1.3] and the branch and foliage equations of [S1.1] with diameter set to the actual diameter (98.0 cm < dbh ≤ 111.0 cm) to a maximum of 111.0 cm (dbh > 111.0 cm).

Abies magnifica (n = 11) – For *Abies magnifica* 1.0 cm ≤ dbh < 30.0 cm dbh (n = 6), we used the generic small conifer proxy [S1.5]. For *Abies magnifica* 30.0 cm ≤ dbh ≤ 109.8 cm (n = 5), we used the total aboveground biomass equation from [S1.2].

Calocedrus decurrens (n = 1,589) – For *Calocedrus decurrens* 1.0 cm ≤ dbh < 2.5 cm dbh (n = 223), we used the generic small conifer proxy [S1.5]. For *Calocedrus decurrens* 2.5 cm ≤ dbh ≤ 165.8 cm (n = 1,366), we used the Cedar/Larch equation from [S1.4].

Cornus nuttallii (n = 2368) – *For Cornus nuttallii* 1.0 cm ≤ dbh < 6.0 cm dbh (n = 1,751), we used the generic small angiosperm proxy [S1.3]. For *Cornus nuttallii* 6.0 cm ≤ dbh ≤ 25.3 cm (n = 617) we used the pooled hardwood equation (maple/oak/hickory/beech) from [S1.4].

Pinus lambertiana (n = 4,746) – For *Pinus lambertiana* 1.0 cm ≤ dbh < 8.7 cm dbh (n = 2,386), we used the generic small conifer proxy [S1.5]. For *Pinus lambertiana* 8.7 cm ≤ dbh ≤ 179.6 cm (n = 2,353), we a bole equation for *Pinus lambertiana* from [S1.3], and branch and foliage for *Pseudotsuga menziesii* from [S1.1], with the branch and foliage equations set to the actual diameter (8.7 cm ≤ dbh ≤ 162.0 cm) or to a maximum of 162 cm (162.0 cm < dbh ≤ 179.6 cm). *For Pinus lambertiana* 179.6 < dbh ≤ 204.1 cm (n = 7), we used a bole equation for *Pseudotsuga menziesii* from [S1.3] and branch and foliage for *Pseudotsuga menziesii* from [S1.1], with the branch and foliage equations set to 162 cm.

Pinus ponderosa (n = 2) – For the *Pinus ponderosa* 1.9 cm dbh, we used the generic small conifer proxy [S1.5]. For the *Pinus ponderosa* 62.8 cm dbh), we used the whole tree equation from [S1.1].

Prunus spp. (n = 128) – For the *Prunus* spp. 1.0 cm ≤ dbh < 6.0 cm (n = 127), we used the generic small angiosperm proxy [S1.3]. For the single *Prunus* sp. 6.0 cm ≤ dbh ≤ 8.2 cm we used the pooled hardwood equation (maple/oak/hickory/beech) from [S1.4].

Pseudotsuga menziesii (n = 6) – For *Pseudotsuga menziesii* 1.0 cm ≤ dbh ≤ 10.2 cm dbh (n = 4), we used the generic small conifer proxy [S1.5]. For the *Pseudotsuga menziesii* 10.2 cm < dbh ≤ 101.5 cm (n = 2) we used the whole tree equation from [S1.4].

Quercus kelloggii (n = 1,109) – For the *Quercus kelloggii* 1.0 cm ≤ dbh < 6.0 cm (n = 273), we used the generic small angiosperm proxy [S1.3]. For *Quercus kelloggii* 6.0 cm ≤ dbh < 59.5 cm (n = 836), we used the pooled hardwood equation (maple/oak/hickory/beech) from [S1.4].

Rhamnus californica (n = 1) – For the 1.1 cm dbh *Rhamnus californica*, we used the generic small angiosperm proxy [S1.3].

Salix scouleriana and *Salix* sp. (n = 11) – For the *Salix* spp. 1.0 cm ≤ dbh < 3.6 cm, we used the generic small angiosperm proxy [S1.3].

Shrub species

Shrub biomass equations were taken from [S1.3].

Arctostaphylos patula – [S1.3; Eq. 736]

Ceanothus cordulatus – substituted equation for *Ceanothus velutinus* [S1.3; Eq. 740].

Ceanothus integerrimus – [S1.3; Eq. 184].

Ceanothus parvifolius – Substituted equation for *Ceanothus integerrimus* [S1.3; Eq. 184].

Chrysolepis sempervirens – Substituted equation for *Alnus sinuata* [S1.3; Eq. 777].

Corylus cornuta var. *californica* – Combined wood/bark and foliage equations [S1.3; Eq. 106 and Eq. 104].

Cornus sericia – [S1.3; Eq. 778].

Leucothoe davisiae – substituted wood/bark and foliage equations for *Vaccinium alaskense* [S1.3; Eq. 133 and Eq. 131].

Rhododendron occidentale – substituted wood/bark and foliage equations for *Vaccinium alaskense* [S1.3; Eq. 133 and Eq. 131].

Ribes nevadense – substituted wood/bark and foliage equations for *Vaccinium alaskaense* [S1.3; Eq. 133 and Eq. 131].

Ribes roezlii – substituted one half the value of the wood/bark and foliage equations for *Vaccinium alaskaense* [S1.3; Eq. 133 and Eq. 131].

Sambucus racemosa – substituted wood/bark and foliage equations for *Corylus cornuta* var. *californica* equations [S1.3; Eq. 106 and Eq. 104].

Vaccinium uliginosum – substituted wood/bark and foliage equations for *Vaccinium alaskaense* [S1.3; Eq. 133 and Eq. 131].

References

S1.1 Gholz HL, Grier CC, Campbell AG, Brown AT (1979) Equations for estimating biomass and leaf area of plants of the Pacific Northwest. Research Paper 41. Forest Research Lab, Oregon State University. Corvallis.

S1.2 Westman WE (1998) Aboveground biomass, surface area, and production relations of red fir (*Abies magnifica*) and white fir (*A. concolor*). Canadian Journal of Forest Research 17: 311-319.

S1.3 Means JE, Hansen HA, Koerper GJ, Alaback PB, Klopsch MW (1994) Software for computing plant biomass – BIOPAK users guide. USDA Forest Service General Technical Report PNW-GTR-340. Pacific Northwest Research Station, Portland, Oregon, USA.

S1.4 Jenkins JC, Chojnacky DC, Heath LS, Birdsey RA (2003) National-scale biomass estimators for United States tree species. Forest Science 49: 12-35.

S1.5 Gower ST, Vogt KA, Grier CC (1992) Carbon dynamics of Rocky Mountain Douglas-fir: influence of water and nutrient availability. Ecological Monographs 62: 43-65.
